# Medicolegal Aspects of Neurosurgery in India: A Narrative Review

**DOI:** 10.7759/cureus.106418

**Published:** 2026-04-04

**Authors:** A Sathia Prabhu, Purbaday Rakshit, Ambika Prasad Patra, George Vilanilam, Vikash Yadav

**Affiliations:** 1 Department of Neurosurgery, Jawaharlal Institute of Postgraduate Medical Education and Research, Puducherry, IND; 2 Department of Forensic Medicine and Toxicology, Jawaharlal Institute of Postgraduate Medical Education and Research, Puducherry, IND; 3 Department of Neurosurgery, Sree Chitra Tirunal Institute for Medical Sciences and Technology, Trivandrum, IND

**Keywords:** court judgements in neurosurgery, medical negligence, medico-legal issues in neurosurgery, neurosurgeon, neurosurgery

## Abstract

Neurosurgery is a surgical specialty that routinely deals with life-threatening pathology that has a narrow therapeutic window and a margin of error. These clinical realities, with most emergencies being medico-legal cases, often expose neurosurgical practice to medico-legal scrutiny. Adequate literature is available on medicolegal aspects of neurosurgery from other countries such as North America, Australia, and Europe. However, literature on medicolegal issues of neurosurgery in India is relatively sparse and fragmented.

Here, we attempt to review the medico-legal aspects of neurosurgery in India in the light of existing legal judgments from Indian courts.

An online search using the keywords “Medical,” “Negligence,” “Neurosurgeon,” “Neurosurgery in India," and their combination was conducted. We found five journal articles available through PubMed using these keywords. Court judgments were screened using the same keywords on Indian legal websites, including Indian Kanoon, AIROnline, and SCC Online[44-46].

After analyzing all the available medicolegal cases through legal websites, we have included 32 cases in this article for discussion. In our study, we found that the doctor was found guilty of negligence in 13 cases (40.6%), while 19 appeals were dismissed (59.4%). The proportion of established medical negligence among cranial cases was found to be 46.7% (seven out of 15), while it was 35.3% (six out of 17) in spinal cases, having a p-value of 0.55. After analyzing the details of all cases, postoperative complications were found to be the major cause for litigation. Lack of adequate informed consent and communication was another common cause.

Neurosurgery is complex and is highly risky; it remains highly vulnerable to medicolegal scrutiny. Strengthening documentation, informed consent, perioperative protocols, and knowledge of medicolegal aspects and their application can save medical professionals from litigation. As per the available literature, this appears to be the first comprehensive review focusing on medicolegal aspects of neurosurgery in India.

## Introduction and background

Globally, the rise in patient awareness, consumer protection laws, and improved access to legal remedies has brought medicolegal issues to the forefront of all medical specialties. In neurosurgery, the inherent complexity, narrow margin for error, and potential for permanent disability or death make adverse outcomes more likely to be challenged legally, both abroad and in India. Developed countries have well-established negligence frameworks, insurance systems, and risk-management protocols, which have shaped strong documentation and consent practices. In India, the Consumer Protection Act and recent National Medical Commission (NMC) regulations have increased scrutiny of medical decision-making, while disparities in infrastructure and workload sometimes hinder optimal documentation and communication. Similar trends are observed in obstetrics, orthopedics, anesthesiology, and critical care, fields where high-risk procedures and unpredictable results create fertile ground for litigation. 

Within the broad field of neurosurgery, spine-related claims usually exhibit a significantly higher rate of filed negligence claims [1]. Common conditions and interventions leading to these claims include spondylolisthesis and laminectomy. Conversely, cranial negligence claims, while less frequent in number, are notably associated with higher litigation compensation awards. This suggests that while spinal issues, often involving chronic pain or mobility limitations, are more prevalent and may lead to frequent patient dissatisfaction, injuries to the brain, which are typically more severe, life-altering, and carry higher morbidity and mortality, result in a greater perceived and legally recognized quantum of harm. This disparity in potential financial liability could influence neurosurgeons' practice to be more cautious or "defensive medicine" in cranial cases due to the higher financial stakes involved.

Existing studies show that [1] the most frequently cited reasons for negligence in neurosurgery include intraprocedural errors, delayed diagnoses, and failure to treat. Other significant allegations encompass misdiagnosis or choice of incorrect procedure, occurrence of death, test misinterpretation, failure to appropriately refer patients for evaluation or treatment, unnecessary surgical procedures, and lack of informed consent. The high percentage of negligence claims is attributed to negligence or miscommunication during the postoperative period. This highlights this phase as a critical vulnerability for neurosurgeons [2]. This implies that while surgical skill is undeniably paramount, effective postoperative management, meticulous monitoring, and clear, empathetic communication are equally vital for preventing litigation and ensuring comprehensive patient care.

## Review

Methodology 

**Figure 1:**
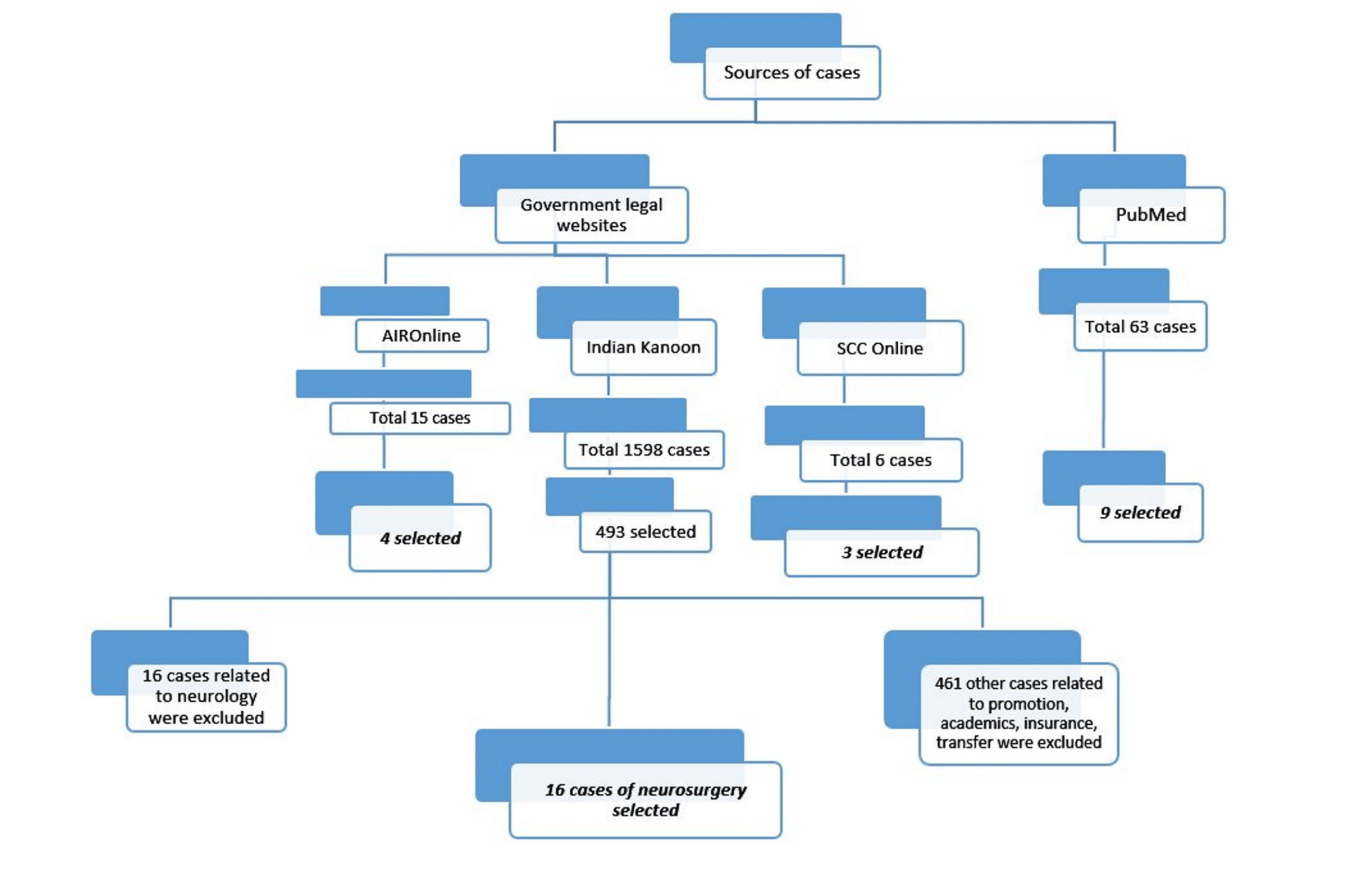
Classification of neurosurgical patients from different sources. n: 32 (AIR Online: 4, Indian Kanoon: 16, SCC Online: 3, PubMed: 9). AIR: All India Reporter, SCC: Supreme Court Cases. Image credit: Created by the authors using Microsoft PowerPoint.

This review of the available data on medicolegal issues in neurosurgery in India and their court judgments was conducted through PubMed and Google using keywords. The judgments were publicly available and accessed through PubMed and Indian legal websites, Indian Kanoon, AIROnline, SCC online, as illustrated in Figure 1 [44-46]. All available judgments to date were analyzed. No human participants were involved in the study. Judgments having the keywords “Medical,” “Negligence,” “Neurosurgeon,” “Neurosurgery in India,” and their combinations thereof were considered. We could find only five journal articles [1-5] through PubMed using the keywords. Our review is based on the cases that we compiled from these articles and the above-mentioned websites. The inclusion criteria comprised all reported Indian court judgments related to neurosurgical practice, both cranial and spinal cases, including cases adjudicated in consumer, civil, and criminal courts. The exclusion criteria included all Indian court judgments not related to neurosurgery, as well as judgments that mentioned neurosurgical conditions or procedures but did not involve a medico-legal dispute or allegation of negligence. The set of relevant data (type of case, diagnosis, surgery performed, compensation asked by the patient, etc.) collected from each judgment is shown. Besides medicolegal cases in neurosurgery, we have also included landmark judgments in other specialties for discussion.

Result

**Figure 2:**
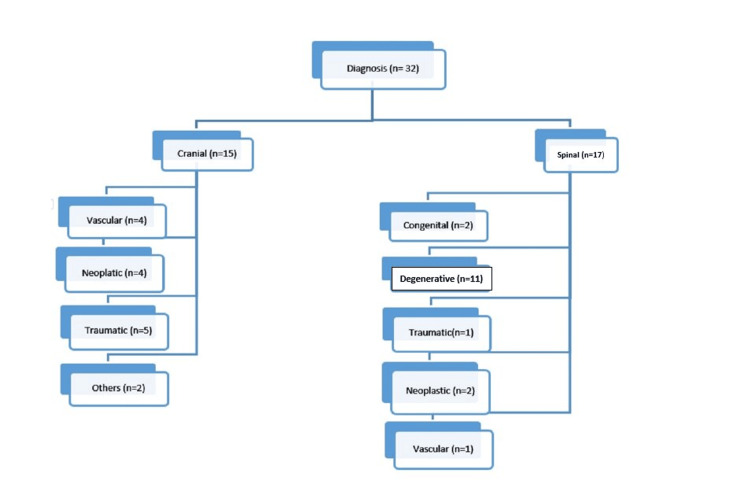
Classification of neurosurgical conditions with which patients presented. Image credit: Created by the authors using Microsoft PowerPoint.

**Figure 3:**
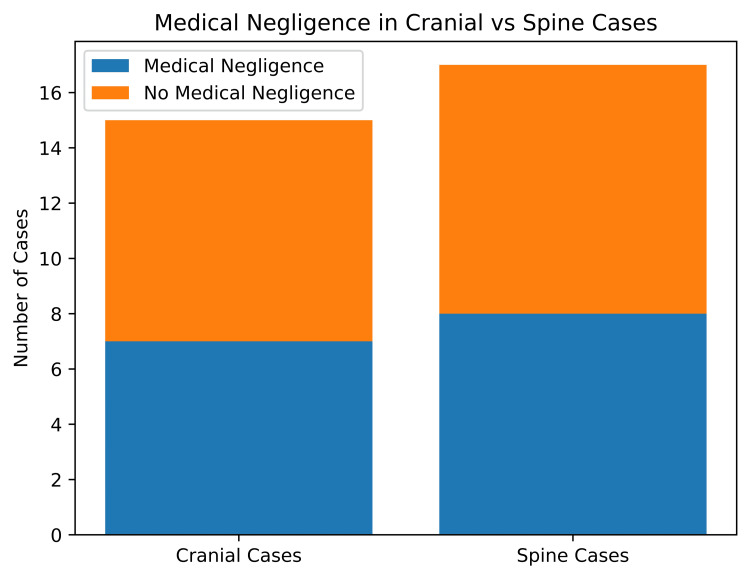
Proportion of medical negligence established among cranial (n=15) and spinal (n=17) cases. Image credit: Created by the authors using Microsoft Excel.

**Figure 4:**
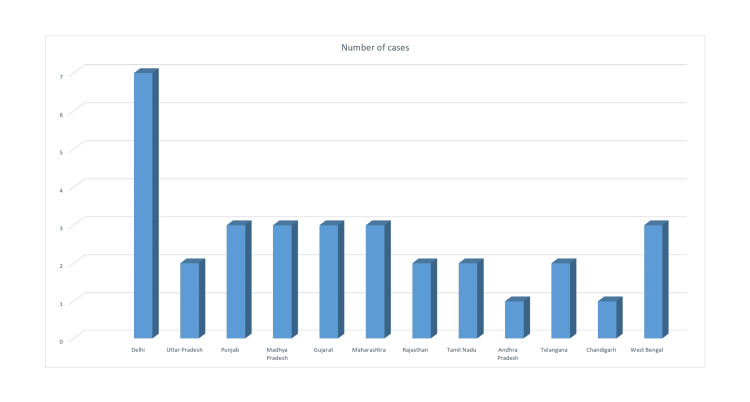
State-wise distribution of medicolegal cases against neurosurgeons in India (a total of 32 cases). Image credit: Created by the authors using Microsoft Excel.

After analyzing all the available cases, we considered 32 judgments for discussion. The doctor was found guilty of negligence in 13 cases (40.6%), while the rest of the appeals were dismissed (59.3%). The proportion of medical negligence among cranial cases was found to be 46.7%, while it was 35.3% in spinal cases, as depicted in Figure 3. Among the cranial cases, post-traumatic cases were the most common (37.5%), followed by vascular (25%) and neoplastic (25%) conditions. Among the spinal cases, the most common was degenerative conditions (56.2%), followed by neoplastic and congenital conditions, as demonstrated in Figure 2. The most common form of case filed in the National Consumer Disputes Redressal Commission (NCDRC) was an appeal against the state Consumer Court judgment (65.6%). Three out of 10 cranial cases operated on showed established medical negligence, while six out of fifteen operated spinal cases showed medical negligence. Postoperative complications were the most common cause of negligence observed (59.3%). Most commonly, the type of intervention done included laminectomy and discectomy among spinal cases (43.75%) overall. Seven out of 13 patients who expired underwent neurosurgical intervention (53.8%). Median compensation awarded by the court was 9.6 lakhs and mean 19.7 lakhs, ranging between a minimum of 25,000 to a maximum of 100,000,000. In 14 out of 32 cases, patients succumbed to their illness (43.7%). Compensation was awarded only in 6 out of those 14 cases (25%). Distribution of medicolegal cases against neurosurgeons across various states of India is shown in Figure 4.

We have selected and analyzed 32 medicolegal cases related to neurosurgery in India, as shown in Table 1.

**Table 1:** List of medicolegal cases specific to neurosurgery discussed in our study. OP: Original Petition, WP: Writ Petition, CC: Complaint Case or Consumer Case, RCR: Revision Case Report, SCC: Supreme Court Cases, AIR: All India Reporter, CPR: Consumer Protection Reporter, CPJ: Consumer Protection Judgments.

CASE	CASE NO	ALLEGATION	COURT VERDICT	CONCERNED ISSUE
Mrs. Kalyani Rajan vs. Indraprastha Apollo Hospital 2023 [11]	OP/74/1999	Inadequate postoperative care in a case of type 2 Chiari malformation who underwent neurosurgery and expired due to ventricular tachycardia raised allegations of inadequate postoperative care	No medical negligence, as complications unrelated to surgery do not amount to negligence	Consent, communication and documentation
Manju Rai vs. Sanjay Gandhi Post Graduate Institute 2022 [12]	OP/409/2002	A case of cervical spondylosis who underwent C-5 and C6 cortectomy, C4-C5 discectomy, OPLL excision, and iliac bone graft placement, expired due to hematemesis, raising the allegation of failure of plating in the initial surgery and unexplained delay in corrective surgery	No medical negligence as a complication was promptly recognized and addressed, not minimized or delayed	Consent, post operative care, detection and management of complications
Mrs Surjeet Sodhi & ORS vs. Fortis Hospital & ANR 2022 [13]	OP/23/2008	A case of bilateral carotid artery thrombosis who underwent carotid stent angioplasty expired due to stroke, raising the allegation that attenders were not informed about risks and possible complications involved with the procedure.	Medical negligence and a compensation of Rs 10,25,000, as the nature and procedure of the treatment and its purpose, alternatives, substantial risks involved, and adverse consequences of refusing treatment were not explained before commencing treatment	Consent about risks and benfits of procedure or surgery
Raj Hospital & Research Centre & ANR vs Mahesh Prasad Varma & ANR 2015 [14]	OP/2/2008	A case of cervical spondylosis with radiculopathy and neck lipoma, who was undergoing conservative treatment for spondylosis, but underwent excision of lipoma, expired due to postoperative bronchospasm	Medical negligence and compensation of Rs 4,50,000, as it was a non‑urgent surgery, there was improper anesthesia, missing consent, and disregard for comorbidities	Consent on risks and benefits of surgery, pre-operative assessment of fitness, necessary investigations
Virendra Singh vs. National Institute of Medical Sciences 2011 [15]	OP/26/2003	A case of Cauda equina syndrome who underwent lumbar laminectomy and discectomy, developed postoperative paraparesis, raising allegations that the patient was operated on by an orthopedic surgeon instead of a neurosurgeon	No medical negligence, as it was a known postoperative complication	Consent, information about operating team
Miss Sunanda G Barve vs Dr Jayant S Barve & Jewel Nursing Home 2018 [16]	CC/00/201	A case of moderate hydrocephalus who underwent a therapeutic lumbar puncture, expired following post-procedure deterioration, raising allegations of the performance of an invasive procedure without consent	Medical negligence with compensation of Rs 5,10,000 because of a lack of informed consent	Consent, risks and complications
Fortis Health Care Ltd vs. Bhagchand Meena 2024 [17]	CC/26/2012	A case of atlantoaxial dislocation that underwent CVJ fixation twice, developed postoperative quadriplegia and expired later, raising an allegation of the absence of any preoperative investigations and the performance of tracheostomy without consent	Medical negligence with compensation of Rs 50,00,000 due to a lack of preoperative workup	Consent, preoperative investigations, communication
Smt Saroj Sharma vs. Government Of National Capital 2019 [18]	Suit No. 614020/16	A case of multiple myeloma involving the fourth thoracic vertebrae that deteriorated following post-myelography convulsion and expired later, raising allegations of a lack of adequate information on complications of an invasive procedure	Medical negligence with compensation of Rs 9,62,888 because of post post-procedure complication	Consent , communication
Smt.Ashwini W/O Shrinivas Pandit vs. Dr.Shyam K.Babhulkar 2022 [19]	CC/16/21	A case of brain tumor who underwent excision, VP shunt, and cranioplasty developed post-operative CSF leak and hemiparesis, raising allegations of post-operative complications	No medical negligence as it these are known complications	Consent about risks and benefits
Vijay Dutt vs. DR R. D. Nagpal & ANR 2014 [20]	OP/199/2001	A case of a bilateral MCA aneurysm that underwent clipping of the left one, who developed postoperative right hemiparesis, raised allegations that the clip was erroneously placed on the MCA vessel itself	No medical negligence as clipping was a preferred treatment for MCA aneurysms, and vasospasm is a known complication causing ischemic stroke leading to hemiparesis	Not all complication is negligence
Smt. Manjulata Garg vs. Dr R.C. Mishra & others 2022 [21]	CC/87/2013	A case of severe osteoporosis with post-traumatic T11 wedge fracture who underwent decompressive laminectomy and transpedicular screw fixation, developed delayed disability, raising allegations of negligence of the surgeon for ignoring osteoporosis	No medical negligence, as postoperative delayed disability is a common complication	Risk and benefit, prognosis
R. Devaraj vs. G. Kuppusamy Naidu Memorial Hospital 2011 [22]	2011 SCC Online TN SCDRC 152.	A case of D9-D10 disc prolapse with cord compression who underwent discectomy and decompression of the cord and re-exploration, developed postoperative paraplegia, raising an allegation of iatrogenic injury during the second surgery, as evident in the MRI scan	Medical negligence with compensation of Rs 5,00,000, as the patient had a poor neurological outcome following the second surgery due to suspected iatrogenic injury	Communication, documentation, complication, avoidable protocols
Dr. Dinesh Chandra Nayak vs. Jaslok Hospital and Research Centre 2017 [23]	CC/03/146	A case of dural arteriovenous fistula undergoing embolization and surgery alleged that the patient underwent an operation at D6 level, though there was an arteriovenous fistula at D8 vertebral level	No medical negligence since although arteriovenous fistula was at D8 level, the supply of blood to it was from the vein at D-6 level which was clipped	Communication, documentation, protoco checklist for complication avoidance
Nuruddin Kutubuddin vs. Dr L.N. Vora 1995 [24]	CPR 424	A case of L5-S1 prolapsed IVD who underwent L5-S1 discectomy had persistence of back pain after surgery, raising allegations of inadequate surgery	No medical negligence, as it was a known postoperative complication and outcome	Communication of prognosis and outcome
H.S.Tuli vs Post Graduate Institute Of Medical Education & Research and others 2008 [25]	CPJ 392 (NC)	A case of a ruptured intracranial aneurysm that expired due to aneurysmal bleeding, raising allegations of deterioration of the patient due to the delay in surgery	No medical negligence, as the nature and outcome of the procedure were explained, but consent was not given by the attenders	Communication, documentation, contributory negligence
G. Ravender Rao vs Ghulam Dastagir 2013 [26]	CPJ 198 (NC)	A case of recurrent primitive neuroectodermal tumor who underwent recurrent excision, expired later, raising allegations of incomplete removal of the tumor during the first surgery	No medical negligence, as the patient expired due to the highly aggressive nature of the tumor	Communication of prognosis
Dr. Rajneesh Goyal vs. Gurpreet Singh 2015 [27]	First Appeal No.409 of 2011	A case of post-traumatic diffuse cerebral edema managed conservatively, raising allegations that the patient deteriorated due to a lack of surgical intervention	No medical negligence, as the patient was managed as per the standard protocol, and the outcome of the condition was explained to the attenders	Communication, documentation, standard management protocol as per guidelines
Nizam’s Institute of Medical Sciences vs. Prasanth S Dhananka & Others 2009 [28]	AIR 2009 SC 1503	A case of mediastinal neurofibroma involving the spine that underwent excision of the tumor along with its spinal extension, developed postoperative paraplegia, raising allegations of lack of neurosurgical assessment and intervention, leading to avoidable spinal cord injury	Medical negligence with compensation of Rs 1,00,00,000 due to a lack of neurosurgical intervention despite preoperative imaging being suggestive of spinal involvement	Preoperative assessment, interdepartmental expert opinion for multidisciplinary involvement if required
Ashit Baran Chakraborty vs. Kasturba Gandhi Hospital 2022 [29]	NCDR Appeal No 74 of 2008	A case of mild traumatic brain injury who was managed conservatively, deteriorated following few episodes of seizures and expired later raising allegations of delay in diagnosis and management	No medical negligence as patient was being treated as per standard protocol and imaging was done to rule out need for urgent surgical intervention	Standard management or protocol guidelines
Shrishti Puri vs AIIMS 2021 [30]	OP/54/2007	A case of congenital kyphoscoliosis who underwent spinal correction surgery by orthopedic surgeon, developed postoperative paraplegia due to alleged spinal screw displacement into the vertebral canal and the absence of supervision by a neurosurgeon	No medical negligence as immediate post-operative imaging was performed in view of anticipated complications, and further management was carried out	Detection and management of post operative complications Qualification of experts
Krishan Lal Kumar vs Medical Council of India & ORS 2023 [31]	W.P.(C)7097 OF 2013	A case of recurrent left sphenoid wing meningioma who underwent tumor decompression expired due to postoperative pulmonary embolism, raising allegations that the patient was operated on without new imaging (MRI scan)	No medical negligence, as new imaging would not have made a difference in the outcome of the disease, and the patient succumbed due to a known post-operative complication	Only essential investigation is appropiate
Vinodkumar Chandanlal Gautam vs State of Gujarat 2024 [32]	R/CR.MA/18663/2014	Case of compressive myelopathy, expired due to pulmonary complications during anesthesia, raised allegations of a lack of proper communication with attenders regarding the patient’s condition	No medical negligence, as the patient succumbed to anesthetic complications before undergoing surgery	Consent, preoperative assessment, risk and benefit
Dr Krishna Mohan Bhattacharjee vs Bombay Hospital & Medical Research Centre 2015 [33]	OP/221/2000	The case of a right recurrent frontoparietal meningioma that underwent surgery thrice, expired after postoperative deterioration, and was comatose, raised allegations of not performing baseline preoperative investigations, such as bleeding and clotting time	Medical negligence with compensation of Rs 10,00,000 as post-operative complications is acceptable, but a lack of basic pre-operative investigations amount to negligence	Protocols and standard in preoperative assessment
Pesarlanka Venkata Subbama vs. SVR Neuro & Trauma Super Speciality Hospital Pvt. Ltd. & ANR 2021 [34]	OP/212/2005	A case of L4-L5 disc prolapse with T12-L1 Koch’s spine, who underwent lumbar laminectomy, has postoperative deterioration, raising allegations of non-performance of investigations for spine tuberculosis before surgery	No medical negligence, but compensation of Rs 50,000 granted	Preoperative evaluation, diagnosis, risk of complications, prognosis
Akhil Bharatiya Grahak Panchayat vs. Dr Jog Hospital 1993 [35]	CPJ 1447	A case of prolapsed lumbar IVD who underwent decompression had no improvement in back pain despite surgery	No medical negligence, as pain can persist postoperatively	Persistence of pain post op does not amount to negligence
Jagrut Nagrik vs. Dr. Yashesh Dalal 2023 [36]	CC/27/2012	A case of L4-L5 PIVD who underwent lumbar discectomy, and laminectomy had developed postoperative infection, raising allegations of negligence against the doctor in ignoring the high blood sugar of the patient	No medical negligence, as there was an acceptable standard of care	Preoperative assessment, postoperative monitoring, documentation of timely intervention
Fortis Escorts Heart Institute vs. Manju Dadu 2024 [37]	CC/326/2012	A case of coronary artery disease with intracranial hemorrhage who underwent coronary angioplasty and evacuation of intracranial hematoma, developed post-operative left hemiparesis, raising allegations of faulty administration of heparin causing intracranial hemorrhage and delay in assessment of ICH	Medical negligence with compensation of Rs 65,00,000, as there was a delay in addressing the complication and subsequent management	Early detection and management of complications
G.S. Sachdeva vs Saroj Hospital & Heart Institute & Ors 2024 [38]	CC/369/2011	A case of obstructive hydrocephalus who deteriorated and got comatose raising allegations of delayed neurosurgical intervention	Medical negligence with compensation of Rs 5,25,000 as deterioration of patient could have been avoided with timely intervention	Delay in intervention
C.R. Gautam vs. Indian Spinal Injuries Centre 2012 [39]	CC/148/2005	A case of severe traumatic brain injury who expired later raising allegations of delay in diagnosis and management	Medical negligence with compensation of Rs 15,00,000 due to established delay in neurosurgical management	Delayed intervention
Jalandhar District Consumer Forum 2021 [40]	CC/408/2014	A case of compressive myelopathy who underwent spinal decompressive surgery developed postoperative neurodeficit, raising allegations of inadequate decompression since the patient worsened after surgery	Medical negligence and compensation of Rs 5,00,000, as there was a lack of adequate documentation, informed consent, and surgical notes.	Consent, communication and documentation, risks of complications, prognosis
Paschim Banga Khet Mazdoor Samiti and Others vs. State of West Bengal 1996 [41]	AIR 1996 SC 2426	A case of post-traumatic intracranial hemorrhage who expired later due to the alleged non-availability of beds in various Government hospitals, leading to a delay in emergency treatment	Medical negligence with compensation of Rs 25,000 due to the non-availability of beds when emergency care was needed	Vicarious liability, lack of infrastructure
Nilam Singh vs. Dr. R.B. Sharma & another 2022 [42]	OP/96/2006	Case of a traumatic acute subdural hematoma who underwent craniectomy and hematoma evacuation, expired later after two surgeries and transfer to another hospital, raising allegations of significant delay in surgical intervention, exacerbating brain damage	No medical negligence as considerable care was taken during two surgeries for primary traumatic SDH and re-accumulation of hematoma, and the patient was transferred to another hospital due to the need for ventilator support	Lack of infrastructure of hospital, post operative complications and management, referral mechanism

Other landmark cases discussed, which are not specific to neurosurgery but relate to general medical practice, include Indian Medical Association vs. V.P. Shantha [6], Jacob Mathew vs. State of Punjab [7], Samira Kohli vs. Dr. Prabha Manchanda [8], Sarwat Ali Khan vs. Prof. R. Gogi [9], and Aruna Ramchandra Shanbaug vs. Union of India & Ors. [10].

Discussion

**Figure 5:**
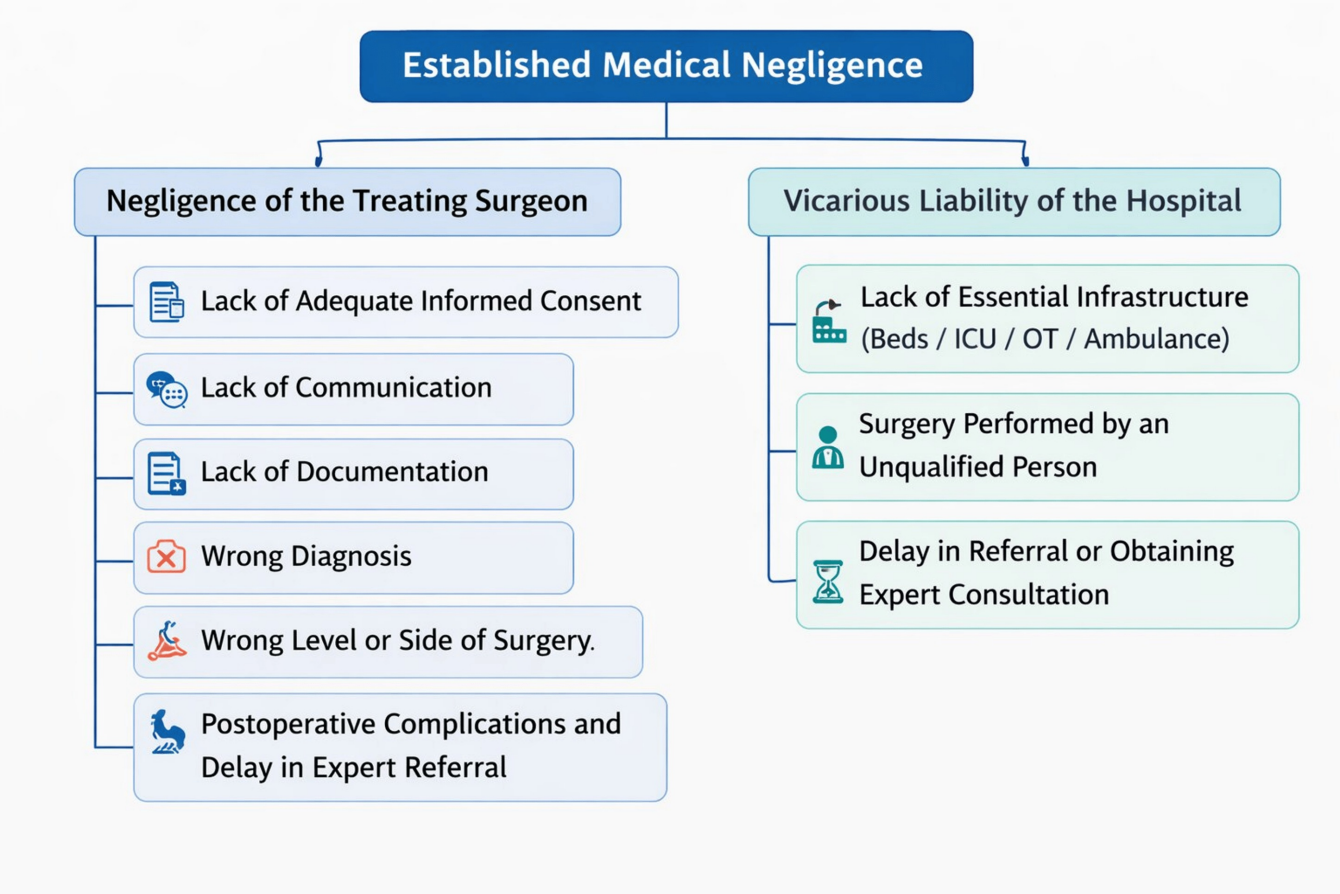
Classification of medical negligence in our study. Image credit: Created by the authors using Adobe Illustrator

Neurosurgery in India has advanced rapidly with improved technology, wider access to care, and rising patient expectations. Alongside these developments, there has been a steady increase in litigation and consumer protection claims against healthcare professionals. The complexity of neurosurgical procedures, the high-risk nature of the specialty, and frequent life-altering outcomes make neurosurgeons particularly vulnerable to medicolegal scrutiny. Courts and consumer forums now demand clear documentation, informed consent, adherence to evidence-based guidelines, and ethical practice. Recent judgments emphasize accountability, transparency, and patient rights, placing greater responsibility on surgeons and institutions. Consequently, awareness of medicolegal principles, risk management strategies, and compliance with national regulations (e.g., NMC guidelines, biomedical waste rules, and telemedicine norms) has become essential for every neurosurgeon. Building a culture of communication, meticulous record-keeping, and continuous legal education is vital to resolving disputes, protecting practitioners, and maintaining public trust in neurosurgical care. Figure 5 illustrates our review of the common causes of established medical negligence in neurosurgery in India. 

Overview of Medical Negligence in India

A medical negligence claim must prove three key elements: the existence of a legal duty to exercise reasonable care, a clear breach of that duty, and damages resulting directly from the breach.

The harm or injury experienced by the patient, whether physical, emotional, or financial, must be proven to be a direct result of the healthcare provider's actions or omissions. It is an important point in Indian law that a mere lack of care, an error in judgment, or an unforeseen accident does not automatically qualify as negligence, provided the medical professional followed acceptable medical standards at the time. The burden of proof is on the complainant rather than the accused.

A victim of medical negligence has several legal remedies: under civil law, the affected person may seek remedy under contract or tort against a medical practitioner for negligence in the performance of professional duties by approaching a civil court or a consumer forum under the Consumer Protection Act; under criminal law, the victim may report the matter to the police or file a complaint before a magistrate under Sections 304A (BNS 106(1)), 337, or 338 of IPC (BNS 125, 126) in cases of gross medical negligence; the victim may also file a writ petition before the concerned High Court under Article 226 or before the Supreme Court under Article 32 of the Constitution of India; additionally, a complaint may be made to the National Medical Commission (NMC).

The legal framework surrounding medical negligence in India has significantly evolved, especially with the inclusion of medical professionals under the scope of the Consumer Protection Act, 1986, following the landmark ruling in “Indian Medical Association vs. V.P. Shantha” [6]. This legislative change enables patients to seek redress and compensation for deficiencies in medical care through consumer forums, including the National Consumer Disputes Redressal Commission (NCDRC).

Private hospitals and nursing homes, corporate and chain hospitals, fall under the consumer court jurisdiction. But it is also applicable for charitable or trust hospitals and government hospitals if they charge any patient even partially. Charitable or trust hospitals and purely voluntary or philanthropic health services are excluded. Only non-government hospitals that provide 100% free treatment to all patients, irrespective of their economic status, are excluded from the Consumer Protection Act. Nowadays, most patients prefer the consumer court due to its easy access, low cost, and early disposals as compared to civil court procedures.

To be included under criminal negligence, there must be gross recklessness in the act causing serious damage to the patient. The Supreme Court's landmark decision in "Jacob Mathew vs. State of Punjab" [7] sets a higher standard for criminal negligence, requiring proof of "gross" or "reckless" conduct that goes beyond simple civil negligence. This legal distinction aims to provide remedies for patients through compensation while protecting doctors from unnecessary harassment and severe criminal penalties, recognizing the inherent risks and uncertainties in medical practice. The court explicitly cautioned against the easy prosecution of doctors without a preliminary expert medical opinion, aiming to curb unjust compensation claims and harassment against medical professionals. Furthermore, the judgment rejected the automatic application of the doctrine of Res Ipsa Loquitur (the thing speaks for itself) in criminal medical negligence cases, [7] emphasizing that the outcome alone does not prove negligence.

Common causes of medical negligence in India

Informed Consent and Communication

The Supreme Court of India, particularly in “Samira Kohli vs. Dr. Prabha Manchanda” [8], has explicitly favored the 'real consent' concept, which aligns with the Bolam and Sidaway principles, over the stricter 'reasonably prudent patient test' inherent in the American 'informed consent' model (Canterbury standard). The judgment underscored that valid consent must be voluntary, provided by a competent patient, and based on adequate information. This preference is deeply rooted in the socio-economic realities of India, where a substantial portion of the population is illiterate or semi-literate and may struggle to comprehend complex medical terminology and treatment procedures fully. Under the 'real consent' framework, the medical professional is afforded a degree of discretion in determining the extent and nature of information regarding risks and consequences that should be disclosed. This discretion is exercised with the understanding that such disclosure must genuinely be in the patient's best interest and align with recognized medical practices.

Key elements of valid consent and disclosure requirements: the three essential elements of consent are that it must be given voluntarily without any coercion, provided by a patient who possesses the requisite capacity and competence to consent, and based on adequate information regarding the nature of the treatment procedure so that the patient clearly understands what they are consenting to.

Disclosure requirements:* *"adequate information” requires disclosure of the nature and procedure of the treatment, including its purpose, benefits, and effects; any available alternatives; substantial risks associated with the treatment; and the potential adverse consequences of refusing the treatment. Written informed consent is mandatory for all invasive procedures, operations, or those involving anesthesia and blood transfusions.

Exceptions to full disclosure:* *the doctrine of therapeutic privilege is an exception to the rule of full disclosure. It permits the withholding of complete information if it is genuinely believed that such disclosure could cause significant harm to the patient, for instance, by frightening an already fearful or emotionally disturbed patient, potentially leading them to refuse necessary treatment despite minimal actual risk. This exception may apply in cases of malignancy or unavoidable fatal lesions, but the risks must still be explained to the family, and the medical professional's rationale for withholding full disclosure is meticulously documented in the patient's record.

In emergencies, where an additional medical procedure, though initially unauthorized, becomes immediately necessary to save the patient's life or preserve their health, and delaying it to obtain explicit consent would be unreasonable, such a procedure is generally permissible.

In the “Samira Kohli vs. Dr. Prabha Manchanda” [8] case, a hysterectomy and bilateral salpingo-oophorectomy were performed without informed consent during a diagnostic laparoscopy for abdominal pain. The patient raised allegations of medical negligence, claiming a lack of informed consent and violation of her right to decide about her own body. The court gave a verdict of established medical negligence.

Consequences of lack of informed consent in neurosurgical cases:* *failure to obtain proper informed consent can serve as a direct basis for a medical negligence claim. While a signed consent form does not guarantee legal immunity, its absence or clear deficiencies certainly create significant medicolegal hurdles for the healthcare provider.

In the case of “Mrs. Kalyani Rajan vs. Indraprastha Apollo Hospital” [11], proper communication with attenders regarding non-surgical postoperative complications was crucial.

The case of “Manju Rai vs. Sanjay Gandhi Post Graduate Institute” [12,13], where a patient had a complication during the first cervical surgery, showed the importance of adequate informed consent mentioning perioperative complications.

In the case of “Raj Hospital & Research Centre & ANR vs. Mahesh Prasad Varma & ANR 2015” [14], the court was unable to locate the informed consent for a non-emergency lipoma excision performed on an elderly patient with significant co-morbidities (asthma, hypertension). The court ultimately held the hospital and doctor liable for negligence, emphasizing that medical professionals are not obligated to abide by patient wishes if it means administering harmful treatment or deviating from standard practice, especially for non-urgent procedures.

However, among recent judgments, the most notable case is "Nizam's Institute of Medical Sciences vs. Prasanth S. Dhananka & Others 2009," where a mediastinal neurofibroma along with its intraspinal extension was excised, rendering the patient paraplegic. This reveals the need for consent for additional interventions.

The case of “Krishan Lal Kumar vs. Medical Council of India," where a patient expired of a pulmonary embolism post-excision of a meningioma, showed the importance of informed consent.

Case of “Smt. Saroj Sharma vs. Government of National Capital 2019" [15-19], where a case of multiple myeloma involving the fourth thoracic vertebrae expired due to post-myelography convulsion, expressing medical negligence due to lack of informed consent.

Comprehensive and Accurate Documentation

Meticulous and accurate recording of all clinical events, actions taken, and patient conditions is a mandatory requirement and a primary defense against litigation [20-27]. This includes detailed medical records, complete and valid consent forms, and thorough documentation of all communication with patients and their families. The increasing trend of obtaining video proof of consent, especially in private healthcare settings, further underscores the importance of robust documentation. This implies that medical institutions should invest significantly in robust electronic health record systems and provide continuous training to their staff on best practices for medicolegal documentation.

Under MCI ethics regulations, indoor patient records must be retained for three years from the start of treatment. For the cases under court procedures, documents need to be retained till disposal of the case.

The case of “Jalandhar District Consumer Forum,” where a patient developed postoperative neurodeficit after spinal decompression surgery, showed established medical negligence due to the absence of adequate perioperative documentation.

The case of “R. Devaraj vs. G. Kuppusamy Naidu Memorial Hospital 2011” [22] showed that a lack of proper procedural documentation amounts to negligence, where the patient developed a neurodeficit after a suspected iatrogenic injury during disc prolapse surgery.

The case of “C.R. Gautam vs. Indian Spinal Injuries Centre 2012," where a patient with a traumatic brain injury expired, showed that there were deficiencies in treatment and gaps in record-keeping, supporting the State Commission’s finding of negligence.

Causation and Proximate Cause

A direct and proximate causal relationship between the alleged breach of duty and the resulting injury is an absolute prerequisite for liability to be established in a medical negligence case [16,21,28,29]. This means the harm suffered must be a foreseeable and direct consequence of the negligent act or omission.

The case of “Nilam Singh vs. Dr. R.B. Sharma & another” serves as a pertinent example. The National Consumer Disputes Redressal Commission dismissed the complaint, concluding that there was no conclusive evidence of medical negligence. The court specifically noted the high mortality rate for the patient's condition (subdural hematoma) and found that the treatment provided adhered to a reasonable standard of care, even when considering the infrastructure limitations prevalent in Patna in 2006. This judgment underscores the necessity of proving a direct causal link between the alleged negligent act and the adverse outcome, rather than merely demonstrating a poor result. This implies that the "standard of care" is not an absolute, idealized benchmark but is rather a contextual one, adapting to the available infrastructure, prevailing medical knowledge, and the time period in which the care was rendered. 

The case of “Smt. Manjulata Garg vs. Dr. R.C. Mishra & others” [21], where a post-traumatic patient with a thoracic vertebra fracture developed delayed neurodeficit, emphasized the fact that a poor surgical outcome is not equivalent to negligence.

The case of “Miss Sunanda G. Barve vs. Dr. Jayant S. Barve & Jewel Nursing Home 2018" [16], where a patient with hydrocephalus deteriorated after undergoing a therapeutic lumbar puncture, emphasized negligence in performing a high-risk invasive procedure without consent or explaining the possible complications. In the case of “Nuruddin Kutubuddin vs. Dr. L.N. Vora” [24] and “Akhil Bharatiya Grahak Panchayat vs. Dr. Jog Hospital," exhibiting persistence of postoperative pain does not amount to medical negligence.

Importance of Preoperative Workup and Perioperative Care

Courts judge negligence by whether a neurosurgeon followed the accepted standard of practice. Preoperative investigations [30-36], imaging, cardiopulmonary evaluation, and anesthesia clearance show that the doctor took reasonable precautions before surgery. Failure to do so can be alleged as negligence. A proper workup identifies comorbidities, bleeding risk, anesthetic risk, etc., and consequently, the family can be warned of the impending risks involved with surgery. Many intra/post-operative catastrophes are avoidable if risks are known, and the court may conclude negligence if a patient dies of a preventable cause. Both a neurosurgeon and an anesthesiologist share responsibility for ensuring patient fitness. In emergencies, as in trauma, a full work-up may not be possible, but minimal investigations like a complete hemogram, coagulation, blood grouping, and an anesthetist’s brief assessment should be documented. Documentation that “life-saving surgery done in emergency without complete work-up due to urgency” protects the neurosurgeon since it is done in good faith for saving a patient's life.

All surgical and medical procedures, especially high-risk ones, must strictly adhere to established standard operative procedures and accepted medical protocols. As per MCI guidelines, OT checklists and SOPs are mandatory to ensure patient safety and minimize surgical errors. The OT checklist verifies patient identity, procedure, site, consent, allergies, anesthesia readiness, instruments, and implants, while SOPs provide standardized protocols for sterilization, infection control, patient positioning, instrument handling, and emergency preparedness. 

In the case of “Dr. Krishna Mohan Bhattacharjee vs Bombay Hospital & Medical Research Centre 2015" [31], re-excision of recurrent meningioma was done, but preoperative bleeding and clotting tests were not done. The court has held the doctor and hospital negligent, as the standard pre-operative workup was deficient.

The case of “Fortis Health Care Ltd. vs. Bhagchand Meena 2024” [17] showed a case of atlantoaxial dislocation that underwent CVJ fixation without preoperative workup, resulting in medical negligence.

It is imperative to perform prompt post-intervention investigations, such as radiological tests, whenever postoperative complications are suspected. This proactive approach can significantly protect medical professionals in a court of law, as demonstrated in cases like “Vijay Dutt vs. DR R. D. Nagpal & ANR” [20], where a repeat angiogram helped validate the defense's actions. This case centered on a dispute regarding the choice of surgical method (clipping versus coiling for a brain aneurysm) and subsequent post-operative complications. The court ultimately dismissed the negligence claim, relying extensively on expert testimony from multiple medical professionals and relevant medical literature. This expert evidence corroborated that the chosen surgical method was justified and that the complications experienced were known risks associated with the procedure.

The case of “Shrishti Puri vs. AIIMS” [30], where the patient developed postoperative paraplegia due to alleged spinal screw displacement, showed the importance of urgent postoperative imaging. *Delay in Diagnosis and Management *

In neurosurgery, delay in diagnosis and management is a major source of medicolegal liability. Most neurosurgical emergencies, like head injury or spinal cord compression, are highly time-sensitive, where even short delays can lead to irreversible deficits or death. Courts interpret such delays as a breach of duty, especially when poor documentation or missing records prevent justification, and often draw an adverse inference against hospitals or surgeons. 

The case of “Fortis Escorts Heart Institute vs. Manju Dadu 2024” [37], where the patient developed an intracranial hemorrhage following coronary angioplasty and subsequently developed hemiparesis post-hematoma evacuation, clearly showed that delay in diagnosis and management amounts to negligence.

In the case of “G.S. Sachdeva vs. Saroj Hospital & Heart Institute & Ors 2024” [38], a patient with obstructive hydrocephalus died without any intervention, showing a delay in neurosurgical intervention amounting to negligence. 

The case of “C.R. Gautam vs. Indian Spinal Injuries Centre 2012” [39] also shows how delayed neurosurgical assessment and intervention caused the death of a severe traumatic brain injury patient. *Surgery on the Wrong Side and Wrong Level*

Both wrong-side and wrong-level surgeries are considered “never events” in neurosurgery. They attract strong legal repercussions like compensation claims, disciplinary action by medical councils, and criminal negligence charges in severe cases [40]. Strict adherence to preoperative verification, intraoperative localization, checklists, and team communication is essential to prevent them. From a medicolegal standpoint, such events are indefensible and usually considered gross negligence. In neurosurgery medicolegal disputes, res ipsa loquitur serves as a powerful tool for patients, as certain adverse events are considered so evidently negligent that courts can infer liability without elaborate proof. 

In the judgment of “Dr. Dinesh Chandra Nayak vs. Jaslok Hospital and Research Centre," the patient was alleged to have undergone surgery at the wrong level (D6) instead of D8, where the arteriovenous fistula was located. However, it was clarified that the blood supply to the fistula came from a vein at D6, which was clipped, nullifying the allegation of wrong-level surgery.

Role of Vicarious Liability in Neurosurgery

In neurosurgery, respondeat superior ensures hospitals and institutions cannot escape liability by blaming only the individual surgeon. It protects patients by holding organizations accountable for system failures and emphasizes the need for institutional risk management, protocols, and supervision.

If a junior neurosurgeon or resident performs a procedure under supervision and commits an error, the supervising consultant may be vicariously liable [41]. Negligence on the part of anesthetists, scrub nurses, neurophysiologists, and radiology staff (e.g., wrong labeling of MRI, anesthesia error, improper instrument sterilization) causes harm; the hospital/lead surgeon can be sued. Written informed consent explaining risks and team involvement; clear documentation of roles (who operated and who assisted); and strict hospital protocols (WHO surgical checklist, marking side/level, and infection control) are some of the preventive measures.

The case of “Mrs. Surjeet Sodhi & ORS vs. Fortis Hospital & ANR 2022” [13] provides a vivid illustration. The District Consumer Disputes Redressal Commission, Kanchipuram, found both the hospital and the consultant doctor negligent. The consultant doctor lacked the requisite qualifications for the intracranial stenting procedure, and the hospital was held liable for appointing him without proper verification of his enrollment with the State Medical Council. This case extends liability beyond individual practitioners to include systemic failures within healthcare institutions, making hospitals vicariously liable for practitioner competence and the integrity of operational and documentation processes.

Lack of Infrastructure in Hospitals

Hospitals bear significant responsibility and can be held liable for engaging medical professionals without thoroughly verifying their qualifications and registration with relevant medical councils. This includes ensuring that appointed practitioners are competent for the specific procedures they are authorized to perform. Healthcare facilities must ensure proper infrastructure, maintain equipment in good working order, and strictly adhere to hygiene and sterility protocols. Lack of an adequate number of ventilators, beds, and ICU setup in a neurosurgical unit hinders the treatment process, as this specialty is involved with a continuous inflow of emergency patients. This indirectly leads to a delay in emergency surgeries and worsening of patient outcomes. This prompts early discharge of sick patients and referral of new patients to other centers having adequate facilities.

The case of “Sarwat Ali Khan vs. Prof. R. Gogi” [9] exposed this lack of infrastructure, where non-functional autoclaves led to hospital liability.

In the case of “Nilam Singh vs. Dr. R.B. Sharma & another” [42], a postoperative traumatic subdural hematoma patient had to be transferred to another hospital due to the need for a ventilator. The patient later expired after transfer to the other hospital.

The case of “Paschim Banga Khet Mazdoor Samiti and Others vs. State of West Bengal 1996" [35] showed how a patient of post-traumatic intracranial hemorrhage expired due to the non-availability of beds at multiple government hospitals, causing treatment delay. The court judgment emphasized that the state shall ensure the availability of adequate facilities at PHC, upgradation of district and subdivisional hospitals, and development of a centralized communication system to ensure bed availability in emergencies.

Other medicolegal issues in neurosurgery

Relevance of Euthanasia in Neurosurgery

Active euthanasia is illegal in India. During the case of “Aruna Ramchandra Shanbaug vs. Union of India 39 & Ors” [10], the Supreme Court has suggested some guidelines for passive euthanasia for terminally ill and non-salvageable patients, which are yet to be made legal in India. Scenarios like severe traumatic brain injury (surviving in a vegetative state), high cervical spinal cord injury (ventilator dependence for life), glioblastoma or end-stage brain tumors (having a very poor prognosis but severe suffering), and post-operative complications with devastating outcomes are common in Neurosurgery. These situations frequently raise requests for withdrawal of treatment, making the need for legislation for passive euthanasia relevant. 

Importance of Court Procedures in Neurosurgery

A neurosurgeon may receive a summons to appear either as a treating doctor or accused (in negligence cases) or as an expert witness (in trauma, assault, or accident cases). Civil court deals with compensation claims for negligence (Consumer Protection Act, NCDRC, State forums), while criminal court deals with rare but possible cases of gross negligence (Section 304A IPC - causing death by negligence). If summoned as an expert witness, one should restrict testimony to facts and medical science and not personal opinion. For a neurosurgeon, a court summons is not a threat but a legal responsibility. 

As per Modi’s book on medical jurisprudence, when doctors are involved in legal cases, they may be summoned to Magistrate, Sessions, or Civil Courts and must appear punctually, take an oath, and give impartial, factual evidence based on their records and findings [47]. Their testimony includes evidence-in-chief, followed by cross-examination and sometimes re-examination, with strict emphasis on clarity, accuracy, and professional honesty. Proper maintenance of medical notes and medico-legal reports is crucial, and doctors are expected to maintain court decorum, address the judge respectfully, and avoid speculation or personal opinions. Wearing a lab coat is not mandatory, but advisable to be easily identified as a doctor in courts.

Role of Telemedicine and Live Surgery in Neurosurgical Practice

Neurosurgeons must be registered with the NMC/State Medical Council to provide teleconsultations. They should abide by Telemedicine Practice Guidelines, 2020 (NMC & MoHFW, India). Misdiagnosis due to poor video quality, lack of physical exam, or missing investigations may still be considered negligence. Neurosurgeons must clearly document limitations of teleconsultation and maintain a telemedicine log mentioning time, advice given, and documents reviewed. Telemedicine is often used for triage in trauma, stroke, and rural settings. Medicolegally, neurosurgeons must advise stabilization and referral to higher centers when appropriate.

For educational purposes, live surgery broadcasts require that strict guidelines be followed to ensure patient safety, confidentiality, and welfare. This includes obtaining explicit, informed, and written consent, ensuring no financial incentives for patient participation, and avoiding the live broadcast of high-risk procedures.

Ghost surgery in neurosurgery

Surgical substitution, or ghost surgery, occurs when a surgeon operates without the patient’s consent, violating informed consent and ethics. This can lead to professional misconduct, disciplinary action, civil liability for negligence, and criminal charges if harm occurs. It is only acceptable in documented team-based surgeries or true emergencies where the designated surgeon is unavailable.

Recent Changes in Bharatiya Nyaya Sanhita (BNS)

The definition of grievous hurt has been modified under BNS (Section 116), replacing IPC (Section 320), as we have shown in Table 2. The required duration of severe pain or incapacity is reduced from 20 to 15 days, with other criteria unchanged. This change could lead to more neurosurgical cases, such as subdural hematomas or postoperative deficits, being classified as "grievous hurt."

**Table 2:** Recent changes in the Bharatiya Nyaya Sanhita (BNS) with corresponding Indian Penal Code (IPC) sections relevant to medicolegal topics IPC: Indian Penal Code, BNS: Bharatiya Nyaya Sanhita Source: Developed by the authors based on review of the literature [43].

Subject	IPC Sections	BNS Sections/ Subsections
Injury	44	2(14)
Causing death by rash or negligent act (medical negligence leading to death)	304A	106(1)
Causing hurt / grievous hurt by rash or negligent act	337, 338	125, 126
Consent, acts done in good faith, benefit of the person	52 (good faith), 80 (accident in doing lawful act), 88 (act not intended to cause death, done by consent), 90 (consent)	26, 30, 18, 19
Definition of grievous hurt	320	116

Under Section 304A IPC, the maximum punishment for all, including doctors, is up to two years of imprisonment, a fine, or both. However, Section 106(1) BNS specifies that registered medical practitioners face up to two years of imprisonment and a fine, while other professional negligence can lead to up to five years of imprisonment and a fine.

Penalties for medical negligence in neurosurgery

Under criminal law (IPC 304A / BNS 106(1)), a practitioner may face imprisonment of up to two years, a fine, or both if death results from gross negligence. Under civil law, courts may award substantial compensation due to the high morbidity and disability associated with neurosurgical cases. Professional misconduct adjudicated by the National Medical Commission or State Medical Council can result in warnings, temporary suspension, or permanent cancellation of the medical license, depending on the severity and recurrence of the negligence. Hospitals or institutions may impose internal disciplinary measures, restrict surgical privileges temporarily, or mandate retraining programs, and they can also face vicarious liability for systemic failures contributing to neurosurgical errors.

Redressal mechanisms for neurosurgeons implicated in medical negligence

Professional bodies such as the National Medical Commission or State Medical Councils provide fair inquiry processes, opportunities for representation, and allow appeals against disciplinary actions. Professional indemnity insurance covers legal defense costs and compensation payouts while providing access to medicolegal experts and advisory teams. Hospital ethics and grievance committees conduct internal reviews of events and may support the surgeon with documentation and expert opinions, facilitating mediation and conflict resolution. Medical associations like the Indian Medical Association, the Neurological Society of India, and state neurosurgical societies offer peer support, medicolegal guidance, expert testimonies, and assistance in mobilizing institutional or legal resources. The legal system, including courts and tribunals, permits the filing of appeals against civil, criminal, or consumer court judgments and ensures due process and protection of the surgeon’s rights. Additionally, documentation and peer review support, including detailed records, operative notes, and multidisciplinary opinions, play a critical role in defending the surgeon in medicolegal proceedings, as we have discussed in Table 3.

**Table 3:** Strategies for neurosurgeons to minimize litigation risk This table is an original compilation created by the authors based on the analysis of judicial judgments from medicolegal cases and is not adapted from any previously published source

Aspect	Do’s	Don’ts
Documentation	Keep clear, dated, complete notes of history, consent, and surgery.	Don’t keep incomplete, vague, or altered records.
Consent	Take valid informed consent in the patient’s language, including risks, alternatives, and prognosis.	Do not rely on blanket consent, and do not skip major risk disclosure.
Protocols	Follow NMC guidelines, WHO checklist, and neurosurgical protocols.	Don’t deviate from accepted standards without justification.
Pre-op Workup	Ensure anesthetic clearance and correct risk factors.	Do not operate without clearance unless in a documented emergency.
Surgical Site/Level	Confirm identity, side, and spinal level with imaging and time-out.	Do not proceed without verification and avoid relying only on memory.
Communication	Give realistic expectations, update family regularly, and disclose complications.	Do not give false assurances, guarantees, and hide complications.
Competence	Operate within expertise, supervise juniors hold mortality review meetings.	Do not attempt complex surgeries beyond your training.
Ethics	Maintain patient confidentiality, avoid unnecessary surgery and refrain from ghost practice	Do not breach privacy or indulge in unethical practices.
Adverse Events	Record and disclose complications factually and manage as per protocol.	Do not conceal events or alter notes after mishaps.
Legal Preparedness	Maintain indemnity insurance, respond to summons, and cooperate with courts.	Do not ignore legal notices or tamper with evidence.

Insurance for neurosurgeons

Indemnity insurance is vital for neurosurgeons because it provides financial protection and legal support against the high risk of litigation. It covers compensation claims and legal fees for defense in civil, consumer, or criminal cases and offers peace of mind, allowing surgeons to focus on patient care. Many hospitals and licensing authorities require such coverage. These policies generally protect against inadvertent errors, but they do not cover willful negligence or criminal acts, making indemnity insurance an essential safety net for professional practice.

## Conclusions

Neurosurgery consistently stands out as a high-risk medical specialty in India, exhibiting one of the highest rates of negligence claims. The high incidence of claims in neurosurgery arises from the inherent complexity and delicate nature of brain and spine surgeries. Even when performed with utmost skill and adherence to established protocols, outcomes can be unpredictable, and complications are an inherent and recognized risk of such procedures. Despite courts frequently ruling in favor of medical professionals in these cases, the sheer volume of complaints suggests a notable disparity between patient expectations, often for perfect or guaranteed outcomes, and the realities of neurosurgical practice. This counseling should extend beyond mere legal compliance to actively manage expectations regarding potential risks, benefits, and the inherent uncertainties associated with neurosurgical interventions, thereby fostering a more realistic understanding of potential outcomes.

This report has provided a comprehensive analysis of Indian court judgments related to the medicolegal aspects of neurosurgery. It has highlighted neurosurgery's inherently high-risk profile for litigation, the centrality of the Bolam Test in determining the standard of care, and the nuanced application of informed consent principles tailored to India's socio-economic context. The increasing apprehension among neurosurgeons, leading to defensive medical practices, underscores the profound impact of the medicolegal environment on clinical care. To navigate this complex landscape effectively, it is imperative for neurosurgeons to prioritize robust medicolegal training, practice meticulous documentation, and foster clear, empathetic communication with patients and their families. Simultaneously, healthcare institutions must uphold stringent standards in credentialing, maintain adequate infrastructure, and implement comprehensive risk management protocols. Striking a sustainable balance between safeguarding patient rights and protecting medical professionals is crucial for fostering a high-quality, trustworthy, and accessible healthcare environment in India. Initiatives for the incorporation of medico-legal education in the regular curriculum of neurosurgery are essential.
